# Evolutionary unraveling: new insights into the *Persicaria amphibia* complex

**DOI:** 10.3389/fpls.2024.1408810

**Published:** 2024-06-26

**Authors:** Gabriella Ballestas, Alexander Nobles, Yoojeong Hwang, Myounghai Kwak, Mi-Jeong Yoo

**Affiliations:** ^1^ Biology Department, Clarkson University, Potsdam, NY, United States; ^2^ Chemistry and Biomolecular Science Department, Clarkson University, Potsdam, NY, United States; ^3^ Strategic Planning Division, National Institute of Biological Resources, Incheon, Republic of Korea

**Keywords:** *Persicaria amphibia*, *Persicaria coccinea*, plastid phylogeny, hybridization, ITS2 haplotypes

## Abstract

The *Persicaria amphibia* complex exhibits significant morphological variation depending on its habitat, existing in either aquatic or terrestrial forms. Traditionally, four distinct elements have been recognized based on morphological features along with their distinct geographical distributions. Recent studies suggest that the Asian element may be genetically distinct from the European and American elements. However, a comprehensive study on the genetic differentiation among all four elements remains lacking. This study aimed to leverage whole plastid genome sequences and ITS2 haplotypes to comprehensively assess the genomic diversity within the *P. amphibia* complex. Notably, we included multiple individuals from New York State to resolve the ongoing debate regarding the taxonomic status of two American elements – whether they represent a single species or distinct entities. Our analysis revealed a well-supported monophyletic clade encompassing all four elements, endorsing their own section, *Amphibia.* Notably, the terrestrial form of the American element is sister to all other elements, suggesting it deserves its own species status. This reinstates its historical name, *P. coccinea*, separating it from the broader *P. amphibia*. Furthermore, distinct compositions of the ITS2 haplotypes differentiated the four elements, although the European element should be further investigated with more sampling. The most intriguing discovery is the identification of putative hybrids between the two American elements. In one population out of four putative hybrid populations, all three entities – the two parent species and their hybrid offspring – thrive together, showcasing a fascinating microcosm of ongoing evolutionary processes. Unraveling the intricate genetic tapestry within each American species and their hybrid populations remains a compelling next step. By delving deeper into their genetic makeup, we can gain a richer understanding of their evolutionary trajectories and the intricacies of their interactions. Finally, it is estimated that the two species of sect. *Amphibia* diverged approximately 4.02 million years ago during the Pliocene epoch, when there was a significant global cooling and drying trend.

## 1 Introduction

Aquatic plants are on the front lines of climate change, such as temperature increases or low precipitation, exhibiting shifting in species composition, a shrinking of their range and distribution, as well as a decline in species richness (Short et al., 2016). Submerged freshwater plants are vital players in the delicate ecosystems they inhabit. They offer refuge, habitat, and a food source for diverse organisms. Their loss would ripple through the entire wetland community, making their proper management and protection a priority for conservation efforts. Identifying distinct species is crucial for effective conservation programs. This knowledge allows us to develop our conservation strategies to meet the specific needs of each vulnerable plant, ensuring their continued survival and the health of the ecosystems they sustain.

*Persicaria amphibia* (L.) Delarbre is an illustrative case of a species whose identity lacks clear consensus within the scientific community. Nonetheless, it holds significant ecological importance within wetland ecosystems. Serving as a primary producer, it provides habitat for various aquatic and semi-aquatic organisms. It contributes to the improvement of water quality by absorbing excess nutrients and filtering pollutants, like many other wetland plants.

**Figure 1:**
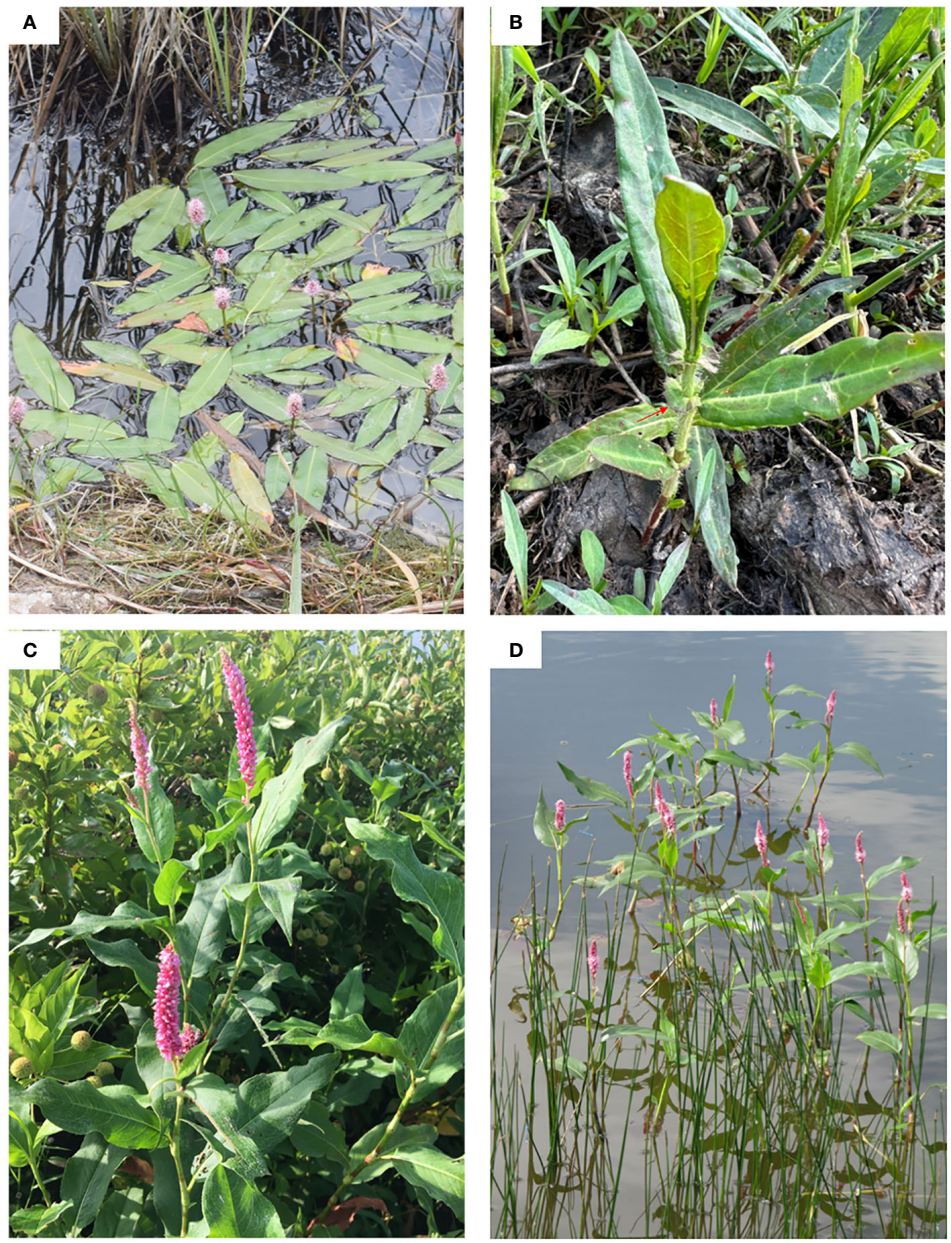
Two North American elements of the *P. amphibia* complex. **(A)** *P. amphibia* var. *stipulacea* (aquatic form), **(B)** *P. amphibia* var. *stipulacea* (terrestrial form), **(C)** *P. coccinea* (= *P. amphibia* var. *emersa*) (terrestrial form), **(D)** *P. coccinea* (aquatic form). Photo credits: **(A)** Nick Belliveau, **(B)** Mi-Jeong Yoo, **(C)** Charley Amos, **(D)** Justin Chappelle.

The *P. amphibia* complex is the most widespread amphibious species within Polygonaceae. It exhibits dynamic morphological transformations depending on where it grows. In aquatic forms, the plants bloom in water with short-cylindric inflorescences (< 4 cm long) (**Figure 1A**). They are glabrous and have long, floating stems with elliptic, obtuse, or rounded leaves and adventitious roots. When they grow on land, they often develop flared ocreae, but this character has only been reported from North American plants (**Figure 1B**) (Mitchell, 1968, 1971). In terrestrial forms, the plants bloom on soil with elongate-cylindric inflorescences (> 4 cm long) (**Figure 1C**). They are often pubescent and have erect or decumbent stems with relatively large linear or ovate, acuminate, strigose leaves and erect roots. In contrast to aquatic forms, they have ocreae that are entire (Mitchell, 1968, 1971; Partridge, 2001; Reveal and Atha, 2010). This terrestrial form can persist under water, but it does not develop floating leaves (**Figure 1D**).

Being native to the northern hemisphere and spanning across Europe, Asia, and North America, this species complex has led to the recognition of four distinct elements based on their morphological features and geographic distribution patterns: 1) The Asian element (var. *amurensis* (Korsh.) H. Hara) characterized by white flowers and cordate leaves; 2) The European element (var. *amphibia*) with pink flowers, which resembles the Asian element; and 3) two different American elements (var. *emersa* (Michx.) J.C. Hickman and var. *stipulacea* (N. Coleman) H. Hara).

In North America, two elements were recognized based on their morphological characteristics; the terrestrial form, *P. amphibia* var. *emersa*, which occurs on soil but does not develop floating leaves when submerged (**Figures 1C, D**), and the aquatic form, *P. amphibia* var. *stipulacea*, which has flared ocreae when stranded on soil (**Figures 1A, B**). However, highly variable morphological transformations were often observed depending on their habitats among these varieties, so their taxonomic entities have been treated differently. For example, Hinds and Freeman (Hinds and Freeman, 2005) integrated these two types into one species without variety ranks. However, Reveal and Atha (Reveal and Atha, 2012) differentiated *P. amphibia* var. *emersa* into a distinct taxon called *P. coccinea* (Muhl. ex Willd.) Greene and maintained *P. amphibia* var. *stipulacea* as it is. In this study, we will use the Reveal and Atha (2012) nomenclature and refer to *P. amphibia* var. *emersa* as *P. coccinea* throughout the paper.

To overcome the limitation of morphological traits in differentiation of this complex, molecular approaches have been taken, which have revealed a clear distinction between *P. amphibia* and other *Persicaria* species (Kim et al., 2008; Kim and Donoghue, 2008a, 2008b). Another study of morphological data of 107 individuals from the *P. amphibia* complex from Asia and North America showed that aquatic forms can be discerned from terrestrial forms by leaf size, shape, and petiole length (Gao et al., 2013). Despite their morphological differences, sequences from four regions of the chloroplast genome failed to distinguish between the two forms, indicating a low level of genetic differentiation between them. In Gao et al. (2013)’s study, 13 individuals from two populations in the United States (California and New York) were included, but only one individual represented the aquatic form. Among the 13 individuals examined, two haplotypes were identified, with a difference of only 16 of 2,760 bp (0.6%). This suggests that the variation in the chloroplast genome within this complex may be very minimal. However, this study focused on a small portion of the chloroplast genome. Therefore, examining both the whole plastome sequences and nuclear regions could provide a more comprehensive understanding of the genetic divergence within this complex. Furthermore, such analysis would help elucidate whether the proposed four elements demonstrate genetic differentiation.

As a polyploid species (2*n* = 66, 88, or 96 for *P. amphibia*, 2*n* = 66 for *P. coccinea*) (Jaretzky, 1928; Sultan et al., 1998), the *P. amphibia* complex typically harbors multiple copies of genes within its nuclear genome, often complicating the analysis of nuclear regions in phylogenetic studies. This applied to the internal transcribed spacer (ITS1 and ITS2) of a segment of nuclear ribosomal DNA (nrDNA). ITS regions have been extensively sequenced and widely employed as a molecular marker in plant systematics due to their valuable features. These include conserved regions suitable for designing universal primers, ease of amplification, and sufficient variability to differentiate closely related species (Coleman, 2003; Schultz and Wolf, 2009; Gao et al., 2010; Pang et al., 2011). Although ITS regions are well known for their homogeneous sequences resulting from concerted evolution, intra-individual polymorphisms in nrDNA have been documented across various plant lineages, including non-hybrid diploids (Xiao et al., 2010; Huang et al., 2016; Xu et al., 2017; Asanuma et al., 2019; Li et al., 2019; Zhang et al., 2022). Thus, the exploration of nuclear markers like ITS regions or single-copy gene areas (e.g., *LEAFY* second intron) requires a laborious cloning process (Kim et al., 2008), often limited to detecting copies occurring in high abundance. Recently, Next Generation Sequencing (NGS) technology has facilitated the direct sequencing of ITS regions at a sufficient depth. This advancement has unveiled significantly higher percentages of intragenomic polymorphisms within eukaryote genomes, including those of plants (Wang et al., 2023).

In this study, we investigated genomic diversity and population differentiation of the *P. amphibia* complex using the whole plastome sequences and the ITS2 region. This study aimed to elucidate the species status of the *P. amphibia* complex in New York State (NY) and tried to answer the following questions: 1) How much genomic variation exists among 12 populations in NY? 2) Are two varieties of this species genetically distinct? 3) If yes, when had these two diverged? 4) Can ITS2 effectively identify these two varieties and their hybrids, if present? 5) Can whole plastome and ITS2 sequences distinguish each element from three regions (America, Asia, and Europe)? The findings of this study will inform updates to the species information in the Flora of North America and provide enhanced insights into the taxonomic status and evolutionary history of this complex.

## 2 Materials and methods

### 2.1 Taxon sampling

To investigate the taxonomic status of the *P. amphibia* complex, leaf tissues were collected from at least ten individuals in each of the 12 populations found in New York (**Table 1**). These twelve populations encompass the two American elements and their putative hybrid populations based on their morphological features (**Table 2**). One individual from each population was selected for DNA library construction, except the Sandy Creek population, where four individuals were chosen. Additionally, one individual from the United Kingdom and two individuals from Asia were obtained from the Royal Botanic Gardens, Kew, and the National Institute of Biological Resources (NIBR; South Korea), respectively (**Table 1**).

**Table 1:** The information of *P. amphibia* complex samples and the population acronyms used in this study.

Species	Population (GPS coordinates)	Acronym	Voucher Information
*P. amphibia* var. *amphibia*	United Kingdom: Somerset	UK	David Gowing, OU 017 (KEW)
*P. amphibia* var. *amurensis*	Korea: Incheon, Ganghwa (37.763472, 126.214953) Russia: Primorsky krai, Khasansky district (42.632192, 130.698669)	Korea 1 Russia	NIBRVP0000749392 NIBRVP0000445926
*P. amphibia* var. *stipulacea*	US: New York: St. Lawrence Co., Watertown (44.008957, -75.883456) US: New York: Saratoga Co., Moreau Lake State Park (43.235554, -73.713526) US: New York: Onondaga Co., Glacier Lake (42.996623, -76.091226) US: New York: Albany Co., Huyck Preserve (42.523163, -74.153782)	WT ML GL HY	Yoo-NY014 Yoo-NY188 Yoo-NY245 Yoo-NY165
*P. coccinea* (= *P. amphibia* var. *emersa*)	US: New York: St. Lawrence Co., Potsdam (44.657782, -74.991088) US: New York: Onondaga Co., Beaver Lake Nature Center (43.180136, -76.407813) US: New York: Saratoga Co., Round Lake (42.939090, -73.789501) US: New York: Saratoga Co., Vischer Ferry Nature and Historic Preserve (42.791611, -73.795411)	PD BL RL VF	Yoo-NY204 Yoo-NY141 Yoo-NY156 Yoo-NY161
Putative hybrid	US: New York: Clinton Co., Lake Alice Wildlife Management Area (44.871501, - 73.483393) US: New York: Franklin Co., Saranac Lake (44.328867, -74.128446) US: New York: Onondaga Co., Guppy Falls Trail (42.970124, -76.392802) US: New York: Oswego Co., Sandy Creek (43.621916, -76.173415)	LA SL GF SC1 SC2 SC3 SC4	Yoo-NY244 Yoo-NY189 Yoo-NY139 Yoo-NY238 Yoo-NY239 Yoo-NY240 Yoo-NY241

**Table 2:** Morphological features of samples used in plastome sequencing.

Population acronym	Inflorescences	Flared ocreae	Leaf shape	Plant height
WT	short, ovoid	Yes	T: lanceolate	< 30 cm tall
ML	short, ovoid	No	A: oblong	< 30 cm tall
GL	short, ovoid	Yes	T: lanceolate	< 30 cm tall
HY	short, ovoid	Yes	T: lanceolate	< 30 cm tall
PD	long, cylindrical	No	T: lanceolate	> 30 cm tall
BL	long, cylindrical	No	T: lanceolate	> 30 cm tall
RL	long, cylindrical	No	T: lanceolate	> 30 cm tall
VF	long, cylindrical	No	T: lanceolate	> 30 cm tall
LA	long, cylindrical	No	T: lanceolate	> 30 cm tall
SL	long, cylindrical	No	T: lanceolate	> 30 cm tall
SC1	long, cylindrical	No	T: lanceolate	> 30 cm tall
SC2	long, cylindrical	No	T: lanceolate	> 30 cm tall
SC3	short, ovoid	No	A: oblong	< 30 cm tall
SC4	short, ovoid	Yes	T: lanceolate	< 30 cm tall
GF	long, cylindrical	No	T: lanceolate	> 30 cm tall

Refer to **Table 1** for the population acronyms. A, aquatic form; T, terrestrial form.

### 2.2 DNA library preparation, sequencing, and assembly

Total DNAs were extracted from silica-gel dried leaves with a DNeasy Plant Mini kit (Qiagen, Hilden, Germany) and quantified using a NanoDrop™ One (Thermo, Waltham, MA, USA). Seventeen DNA libraries (two Asian individuals, one European individual, and 14 American individuals) were constructed with 100 ng of DNA using the NEBNext^®^ Ultra™ II DNA Library Prep Kit for Illumina^®^ (New England Biolabs, Ipswich, MA, USA). Each library was indexed with multiplex oligos and quantified with the NEBNext Library Quant Kit (New England Biolabs, Ipswich, MA, USA). After quantification, equimolar libraries (5 nM each) were pooled together and sequenced with 2 ×150 bp on an Illumina NovaSeq 6000 (Illumina, San Diego, CA, USA) at the Interdisciplinary Center for Biotechnology Research (ICBR) of the University of Florida. The obtained sequences were cleaned with Trimmomatic v. 0.36 (Bolger et al., 2014) and assembled into the plastome sequences and nuclear ribosomal DNA (nrDNA) sequences using GetOrganelle 1.7.5.0 (Jin et al., 2020). The assembled sequences were then confirmed with Geneious v.2024.0.1 (http://www.geneious.com).

### 2.3 Sequence alignment and phylogenetic analysis

Assemble plastome sequences were annotated using GeSeq (Tillich et al., 2017) and visualized with OGDRAW (Greiner et al., 2019). These sequences were aligned with MAFFT v.7.520 (Katoh and Standley, 2013) and manually adjusted. Phylogeny was inferred with the maximum likelihood (ML) method using IQ-TREE v.2.2.2.7 (Nguyen et al., 2015). For the ML tree, molecular evolution models were evaluated using ModelFinder (Kalyaanamoorthy et al., 2017), and the standard nonparametric bootstrap method (-b 1000) was applied to evaluate the support of retrieved clades. Plastome sequences from members of sections *Persicaria*, *Tovara*, *Echinocaulon*, and *Cephalophilon* were retrieved from NCBI GenBank. The sequences of sect. *Cephalophilon* were utilized as outgroups in the analysis.

### 2.4 Genotyping population using two chloroplast regions

To evaluate the origin of putative hybrid populations and genetic differentiation within and between populations, two variable regions of the plastome between *P. amphibia* var. *stipulacea* and *P. coccinea* were investigated. Out of seven intergenic spacers (IGS) and intron regions analyzed, we chose two regions that showed the most substantial size difference (**Supplementary Table 1**). Two target areas were amplified in a 50 µL PCR reaction that included 5 – 50 ng of DNA, 0.5 µM of each primer, and 25 µL of OneTaq^®^ 2× Master Mix (New England Biolabs, Ipswich, MA, USA). The PCR was performed as follows: 3 minutes of initial denaturation at 95°C, followed by 35 cycles of 30 seconds at 95°C, 30 seconds at 55°C, 45 seconds at 72°C, and the final extension of 5 minutes at 72°C. The amplicons were assessed using a 1.2% agarose gel, running at 100 V for 30 min.

### 2.5 ITS2 haplotype identification

To identify ITS2 haplotypes, we initially aligned short reads to the ITS regions and assessed sequence variations. Given that ITS2 showed more variability than ITS1, we extracted representative haplotypes from each sequencing dataset. Subsequently, 133 bp of ITS2 haplotypes were tallied from the short reads of each DNA library using a custom script. During this process, short reads that fully encompassed the entire 133 bp of ITS2 were counted, and the results were presented as a pie chart.

### 2.6 Divergence time estimates

The divergence times of the *P. amphibia* complex were estimated using BEAST v.2.6.2 (Bouckaert et al., 2019) based on a plastid ML tree. First, BEAST input XML file was generated using BEAUti v2.7.5. A GTRGAMMA evolutionary model was used with 10 gamma rate categories. A log-normal relaxed clock (Drummond et al., 2006) was the clock model, using the ML tree estimated above as the starting tree, and with speciation modeled by the Yule process. Age calibrations were implemented as follows: a log-normal distribution with a mean of 1.0, a standard deviation of 1.0, and an offset of 31.14 for the crown group of sections *Persicaria*, *Tovara*, and *Echinocaulon*, and an offset of 14.29 and 17.39 for sections *Cephalophilon* and *Echinocaulon*, respectively (Cao et al., 2022). We ran five separate runs for a total of 50 million generations, sampling every 1000. Runs were combined using LogCombiner v.2.7.5 with a 10% burnin, and a maximum clade credibility (MCC) tree with mean node heights was created using a sample of 18,002 trees in TreeAnnotator v.2.7.5. The chronogram was drawn using FigTree v.1.4.3 (http://tree.bio.ed.ac.uk/software/figtree/).

## 3 Results

### 3.1 Assembled nrDNA and plastome sequences

Assembled nrDNA is about 5,868 bp, but there was little sequence variation (0 – 10 bp differences among the *P. amphibia* complex; data not shown). In the ITS region of nrDNA, the Asian and European elements have identical sequences with some gaps, while the American elements showed a difference of seven out of 750 bp compared to the Asian and European elements. Thus, due to their low sequence divergence and heterogeneity, nrDNA sequences were not employed in phylogenetic analysis. Instead, a thorough investigation was conducted on the compositions of ITS2 haplotypes (refer to the information below).

Assembled plastome sequences range from 159,320 bp (*P. amphibia* var. *stipulacea* from Sandy Creek) to 159,539 bp (*P. coccinea* from Vischer Ferry) and have a typical structure of a plastid genome with two inverted repeats (IRA, IRB), which separate a long single copy section (LSC) from a short single copy section (SSC). However, the assembled plastomes showed no structural variations among the individuals investigated here (**Supplementary Figure 1**). There are 79 protein-coding genes, 4 rRNA genes, and 30 tRNA genes arranged in the same gene order (**Supplementary Figure 1**).

### 3.2 Phylogenetic analysis of plastome sequences

**Figure 2:**
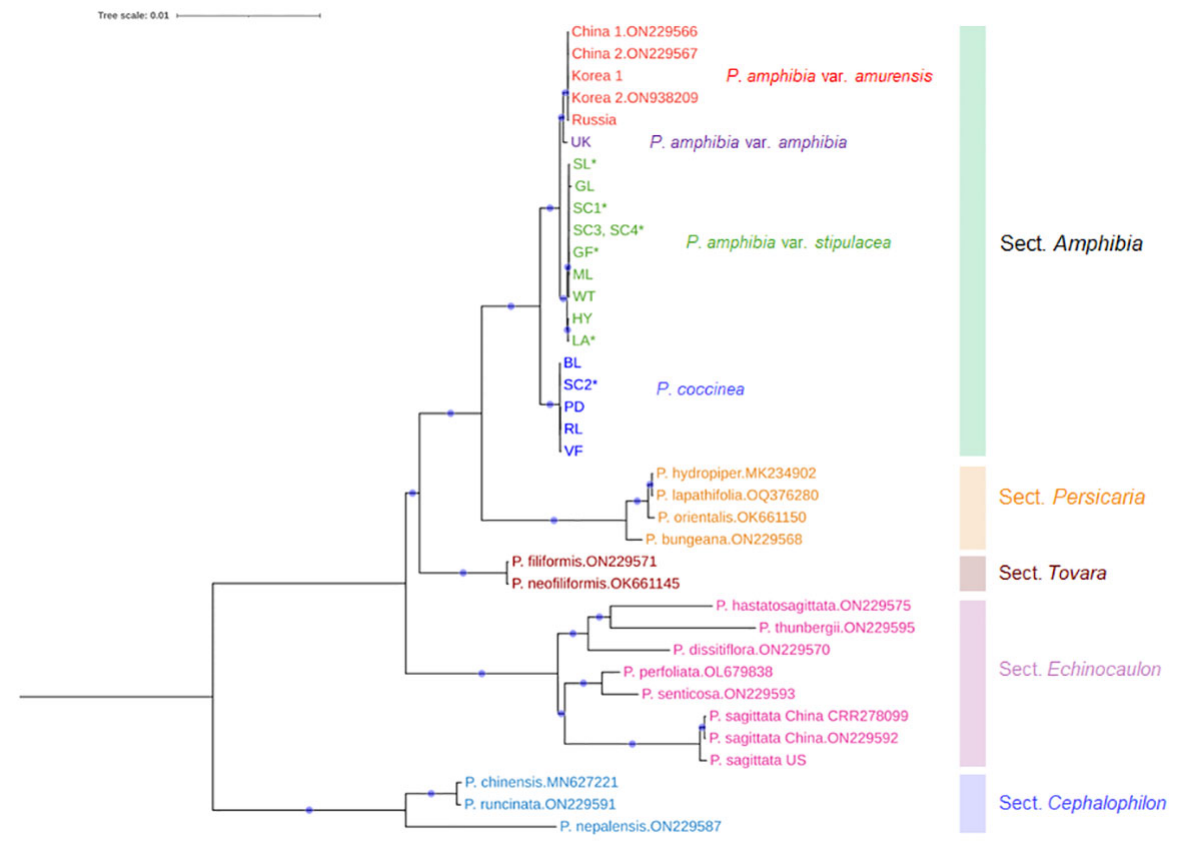
A maximum-likelihood tree inferred from the entire chloroplast sequences with the best fit model of K3Pu+F+R2. Four elements were indicated by different colors. Red: Asian element, purple: European element, green: American element I, blue: American element II. The aligned length was 168,017 bp, of which 90.8% (152,514 bp) were constant across taxa and 6.8% sites (11,365) were parsimony informative. The navy circle on the branch shows 100% bootstrap support value. Asterisk (*) indicates putative hybrids. For sequences downloaded from NCBI, their accession numbers were presented next to the species name.

The analysis of plastome sequences revealed a strongly supported monophyletic clade that includes all four elements, endorsing their own section, *Amphibia*, as well as confirming the monophyly of each section of *Persicaria* (**Figure 2**). However, the American elements failed to form a single clade: the American element I (*P. amphibia* var. *stipulacea*) was closely placed in the clade containing the Asian and European elements, while the American element II (*P. coccinea*) was sister to the clade that includes the other three elements (**Figure 2**). The genetic distance between American element II and the other three elements was much greater compared to those observed among the members of sect. *Persicaria* and *Tovara*. Thus, American element II should be considered a distinct species, *P. coccinea*.

**Figure 3:**
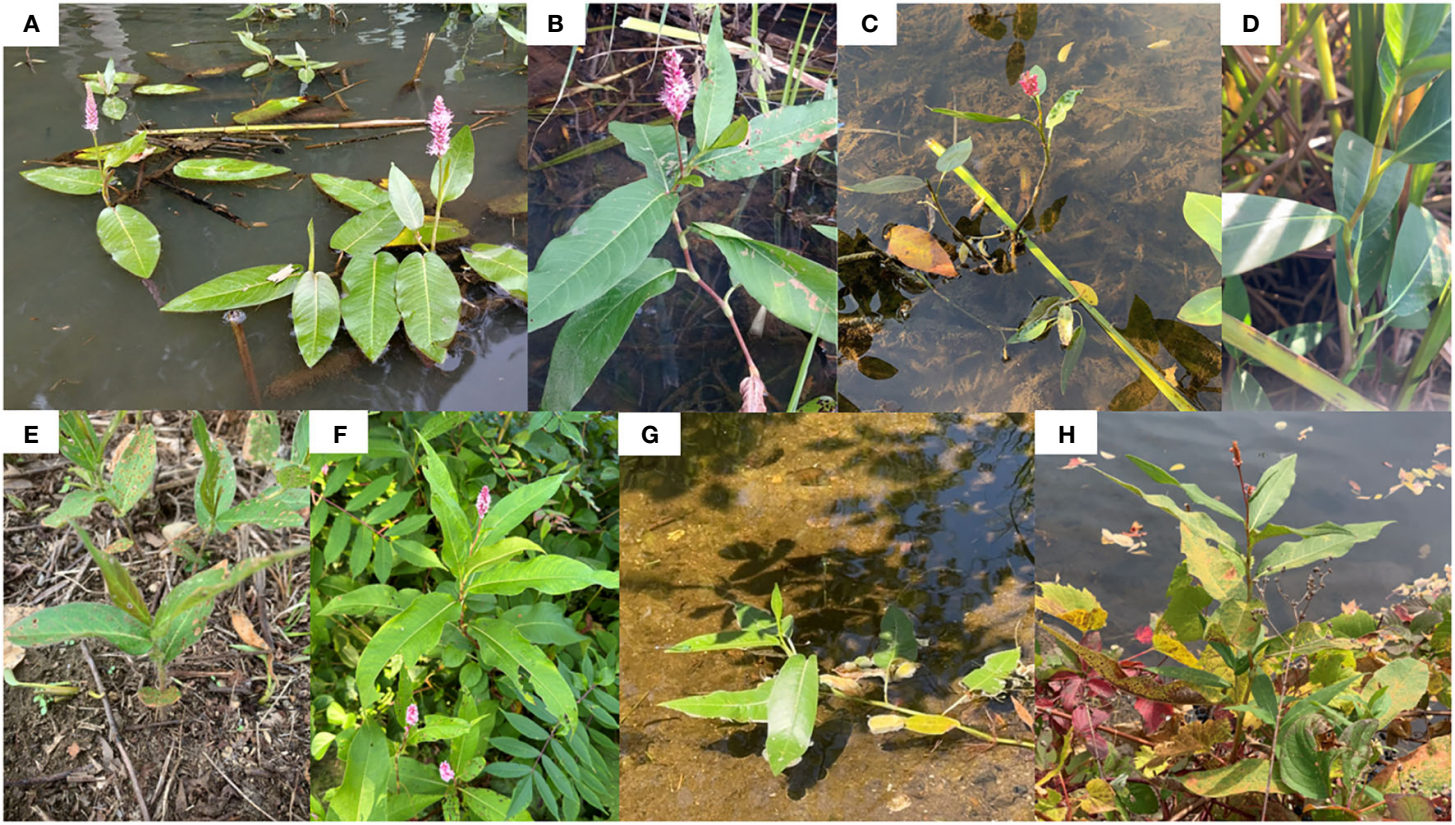
Putative hybrids from four populations. **(A)** *P. amphibia* var. *stipulacea*-like aquatic form and **(B)** *P. coccinea*-like terrestrial form from Guppy Falls (GF); **(C)** *P. amphibia* var. *stipulacea*-like aquatic form and **(D)** *P. coccinea*-like terrestrial form from Lake Alice (LA); **(E)** *P. amphibia* var. *stipulacea*-like terrestrial form and **(F)** *P. coccinea*-like terrestrial form from Sandy Creek (SC); **(G)** *P. amphibia* var. *stipulacea*-like aquatic form and **(H)** *P. coccinea*-like terrestrial form from Saranac Lake (SC). Photo credits: **(A, B)** Randy A. Nonenmacher, **(C–H)** Mi-Jeong Yoo.

Interestingly, all putative hybrid species were included in the clade of *P. amphibia* var. *stipulacea*, except one individual (SC2) from Sandy Creek (**Figure 2**). This individual is morphologically similar to that of *P. coccinea*, while the other three individuals (SC1, SC3, and SC4), containing the plastome of *P. amphibia* var. *stipulacea*, exhibit similarities to *P. coccinea* and *P. amphibia* var. *stipulacea*, respectively (**Tables 2**, **3**; **Figure 3**). Thus, there might be all three taxa, two parental species and their putative hybrid species, occurring together in Sandy Creek (see below).

**Table 3:** Comparison of morphological and molecular characteristics of putative hybrids.

Individual	Morphology	Plastome	ITS2 haplotypes
GF	*P. coccinea* (**Figure 3B**)	*P. amphibia* var. *stipulacea*	*P. coccinea* (northern type)
LA	*P. coccinea* (**Figure 3D**)	*P. amphibia* var. *stipulacea*	*P. coccinea* (northern type)
SL	*P. coccinea* (**Figure 3H**)	*P. amphibia* var. *stipulacea*	*P. coccinea* (northern type)
SC1	*P. coccinea* (**Figure 3F**)	*P. amphibia* var. *stipulacea*	*P. coccinea* (northern type)
SC2^*^	*P. coccinea* (**Figure 3F**)	*P. coccinea*	*P. coccinea* (southern type)
SC3	*P. amphibia* var. *stipulacea* (**Figure 3E**)	*P. amphibia* var. *stipulacea*	*P. coccinea* (northern type)
SC4^*^	*P. amphibia* var. *stipulacea* (**Figure 3E**)	*P. amphibia* var. *stipulacea*	*P. amphibia* var. *stipulacea*

Asterisk (*) indicates potential parental species in the Sandy Creek population.

In the meantime, the European element was placed as a sister to the Asian element in the clade of *P. amphibia* (**Figure 2**), but its branch length was too short, and its sequence divergence was too low (0.06%) to differentiate it from the Asian element. Therefore, we analyzed the partial chloroplast sequences of a Greek individual from the previous work (Gitsopoulos et al., 2013) alongside the current data to determine the differentiation between the European and Asian elements. While the monophyletic clades of two species, *P. amphibia* and *P. coccinea*, were retrieved in agreement with the ML tree inferred from the entire plastome data, two individuals from Europe (UK, Greece) failed to form their own clade (**Supplementary Figure 2**).

### 3.3 Genotyping populations using two chloroplast regions

Based on the chloroplast sequences, we designed primers targeting the *rpl20*-*rpl12* IGS and *rpl33-rps18* IGS, which yielded different-sized fragments between two American elements. Utilizing the regions, we conducted screenings of individuals from 12 populations. Based on morphological features, four hybrid populations were identified. The plants in four hybrid populations exhibited a morphological resemblance to *P. coccinea*. However, noteworthy variations were observed; for example, certain individuals developed floating leaves when grown in water and exhibited flared ocreae when stranded on dry land (**Table 2**; **Figure 3**).

The screening results unequivocally confirmed the identities of *P. amphibia* var. *stipulacea*, *P. coccinea*, and their hybrids (**Supplementary Figure 3**). Of particular interest, the Sandy Creek population displayed the co-occurrence of two plastomes, while other hybrid populations contained only the plastome of *P. amphibia* var. *stipulacea* (**Supplementary Figure 3**). Additionally, it is worth noting that most hybrids possess a plastome that is similar to the American element I (*P. amphibia* var. *stipulacea*). This suggests that *P. amphibia* var. *stipulacea* may have been the maternal parent during the hybridization events.

### 3.4 ITS2 haplotype analysis

**Figure 4:**
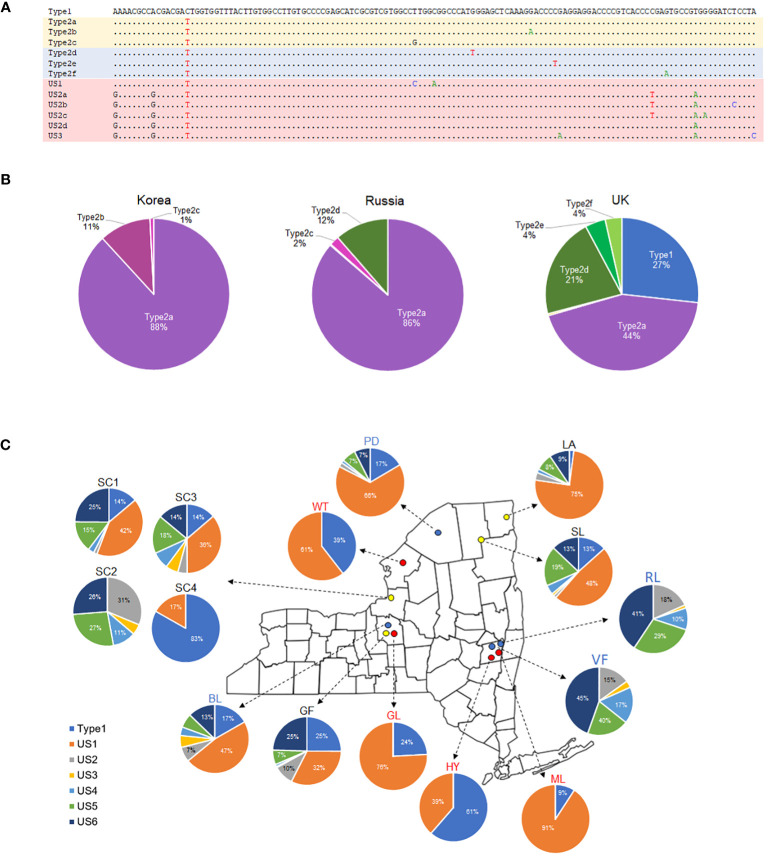
ITS2 haplotypes sequenced in the *P. amphibia* complex. **(A)** Sequences of ITS2 haplotypes, **(B)** pie charts showing the compositions of ITS2 haplotypes in *P. amphibia* var. *amurensis* (Korea, Russia) and *P. amphibia* var. *amphibia* (UK), **(C)** Pie charts showing the compositions of ITS2 haplotypes in *P. coccinea* (blue circles), *P. amphibia* var. *stipulacea* (red circles), and their putative hybrids (yellow circles) collected from New York, USA.

To find out how many different ITS2 haplotypes exist, short reads were assembled into nrDNA and examined manually. The study revealed the presence of three ITS2 haplotypes in the Asian element, five in the European element, and seven in the American elements (**Figure 4A**). Then, the frequencies of each haplotype were determined based on around 1000 short reads, that provided complete coverage of the 133 bp region of the ITS2 haplotype. As a result, the Type2a haplotype accounted for 88% and 86% of the ITS2 haplotypes in individuals from Korea and Russia, respectively. In the European element, the Type1, Type2a, and Type2d haplotypes together represented 92% of the ITS2 haplotypes (**Figure 4B**). The composition of ITS2 haplotypes in the American elements exhibited variations. Specifically, four individuals of *P. amphibia* var. *stipulacea* (GL, HY, ML, and WT) and one individual from Sandy Creek (SC4) were found to have just two haplotypes (Type1 and US1). On the other hand, *P. coccinea* and putative hybrids had a higher number of haplotypes, ranging from five to seven (**Figure 4C**). In addition, the geographical differences were evident in the composition of ITS2 haplotypes in *P. coccinea*. Among the *P. coccinea* individuals studied, two individuals from the southern region (RL, VF) and one individual from Sandy Creek (SC2) were found to lack Type1 and US1, which are prominent haplotypes observed in the remaining individuals. Furthermore, three distinct compositions of ITS2 haplotypes were identified in the Sandy Creek populations: 1) SC1 and SC3 exhibit similarities to northern *P. coccinea* (PD and BL), 2) SC2 shows similarity to southern *P. coccinea* (RL and VF), and 3) SC4 displays characteristics comparable to *P. amphibia* var. *stipulacea* (**Figure 4C**).

### 3.5 Divergence time estimates

**Figure 5:**
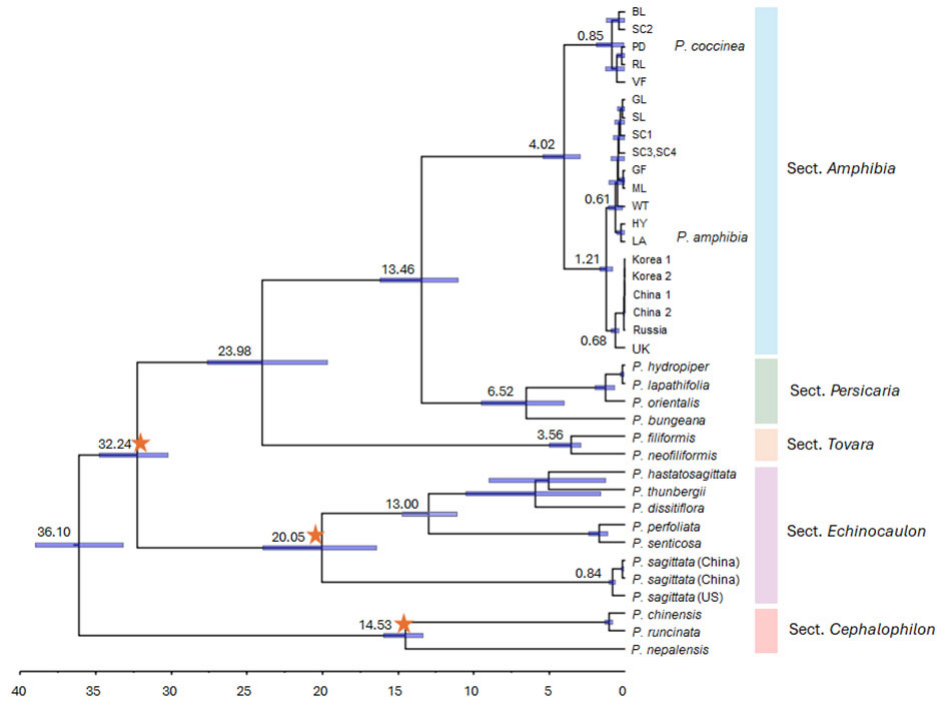
Divergence time estimates for *Persicaria* species inferred from the whole plastome sequences. Blue bars represent 95% highest posterior density (HPD) intervals. Orange stars show the calibration points used in this study.

To estimate divergence times for the *P. amphibia* complex, we utilized the ML tree obtained above and three calibration points that were reported previously (Cao et al., 2022). The estimated divergence times for lineages exhibited variation in comparison to the previous work based on 80 plastome sequences of Polygonaceae (Cao et al., 2022). For example, the genus *Persicaria* diverged 36.10 million years ago (Ma) (95% HPD: 33.18–39.01 Ma) in this study, which was younger than 42.78 Ma (95% HPD: 34.72–45.36 Ma) in the previous work of Cao et al. (2022) (**Figure 5**). However, the 95% HPD for the major clades showed overlap with the 95% HPD of Cao et al. (2022)’s estimates.

Based on our analysis, we determined that sections *Persicaria* and *Amphibia* diverged approximately 13.46 Ma (95% HPD: 11.01–16.21 Ma), while two members of sect. *Amphibia* split apart around 4.02 Ma (95% HPD: 2.95–5.42 Ma) (**Figure 5**). Finally, *P. amphibia* var. *stipulacea* diverged approximately 1.21 Ma from other elements.

## 4 Discussion

### 4.1 Support of section *Amphibia* and two distinct species in American elements

The genus *Persicaria* has about 100 species and can be further classified into four sections: *Persicaria*, *Tovara*, *Echinocaulon*, and *Cephalophilon* (Haraldson, 1978). Among them, sect. *Persicaria* stands out with the highest number of taxa. Notably, the *P. amphibia* complex has long resided within this section. However, recent molecular evidence (Kwak et al., 2006; Kim and Donoghue, 2008b; Cao et al., 2022) and their unique morphological features, such as pollen typology (Kong et al., 2021) and the presence of the rhizome, placed this complex as its own section of *Amphibia*. Nevertheless, the previous investigations failed to examine all four elements of the *P. amphibia* complex. In this study, we incorporated all four elements for the first time to elucidate the species entities and their relationships.

The *P. amphibia* complex has been well known for its high polymorphisms, depending on its environment. This variability had led to many synonyms; for example, 187 names were listed in Plants of the World Online (POWO). However, it has long been recognized that the American elements were clearly differentiated from Asian or European elements as they possessed flared ocreas when stranded. A recent analysis of nearly 10,000 observations on iNaturalist shed further light on this taxonomic tangle by confirming a clear morphological distinction between the two American elements, although there were a handful of exceptions (less than 0.1% of all observations) (Atha, 2024). Therefore, Atha (2024) proposed the existence of two species, *P. amphibia* var. *stipulacea* and *P. coccinea*, within the American elements. However, no molecular data has been shown to support this claim.

Here, we delved deep into the plastome sequences of the American elements, seeking to establish their species entity. Furthermore, we included Asian and European elements to evaluate whether they could be genetically differentiated from the American elements. We assembled 17 plastome sequences, and upon analysis, it was seen that four elements were clustered together, receiving 100% bootstrap support (**Figure 2**).

In the *Amphibia* clade, there were remarkable genetic similarities among four individuals of the Asian element, while the European individual exhibited slight genetic differentiation from the Asian individuals (**Figure 2**). Even though the European element was sister to the clade of the Asian element (**Figure 2**), it is important to examine this element more thoroughly because of its wide distribution in Europe and the failure to form a monophyletic group in the analysis of two European individuals (**Supplementary Figure 2**). In addition, there were some shared features in the ITS2 haplotypes between Asian and European elements, such as Type2a and Type2d (**Figure 4B**). These results indicate that further investigation with more samples is required to confirm the differentiation of the European element from the Asian element and its taxonomic status. In contrast, the American elements formed two separate clades. The first one included all the aquatic forms, along with some terrestrial forms, and is closely related to a clade consisting of the Asian and European elements (**Figure 2**). All the aquatic forms (HY, GL, ML, and WT) have typical morphological features of *P. amphibia* var. *stipulacea*, such as ovoid and short inflorescence, oblong leaves, short plant height, and flared ocreae when stranded. In addition, they had only two types of ITS2 haplotypes, Type1 of which was shared with the European element (**Figures 4B, C**), supporting their close relationship as shown in the plastome phylogeny (**Figure 2**). Therefore, based on their consistent morphology and plastome sequences, the members of this clade are classified as *P. amphibia* var. *stipulacea*.

The other clade contains terrestrial forms of *P. coccinea*-like plants only and is sister to the other elements (**Figure 2**). This group, genetically separated from the other elements by 0.32 – 0.34% in the whole plastome sequences (twice as high as that between *P. orientalis* and *P. lapathifolia*), deserves its own species recognition. Also, the members of this clade (BL, PD, RL, and VF) had five to seven different types of ITS2 haplotypes (**Figure 4C**). Thus, the American element II should be recognized as a distinct species, *P. coccinea*, based on its unique morphology and significant genetic differentiation from the other elements.

As a result, the phylogenetic analysis of plastome sequences strongly corroborates sect. *Amphibia*, which comprises two species, *P. amphibia* and *P. coccinea* (**Figure 2**). Notably, the morphological and molecular characteristics of the full plastome sequences and ITS2 haplotype composition provide evidence for the existence of two distinct species in American elements: *P. amphibia* var. *stipulacea* and *P. coccinea*. This suggests independent evolutionary trajectories for these distinct species (**Figure 4C**). Specifically, we estimated that their divergence occurred 4.02 Ma (**Figure 5**), during the Pliocene epoch when there was a significant global cooling and drying trend. This suggests that their distinct environmental preferences may have influenced their evolutionary process over a period. For instance, despite both species being amphibious, these species exhibit differences in population size depending on their habitat. *Persicaria amphibia* forms significantly larger populations in aquatic environments compared to terrestrial habitats, suggesting a better adaptation to such habitats. Conversely, *P. coccinea* prefers terrestrial habitats but can tolerate temporary flooding. These adaptations are reflected in their distribution patterns: while both species are found throughout North America, *P. amphibia* var. *stipulacea* is absent south of the Laurentide Ice Sheet, where aquatic habitats would differ from those to the north of it (Atha, 2024).

### 4.2 Hybridization between Two American Elements

As Atha (2024) noted, there are several reports on American elements that have intermediate phenotypes between *P. amphibia* var. *stipulacea* and *P. coccinea*. We also identified four populations that display intermediate morphological characteristics (GF, LA, SC, and SL; **Table 2**).

Interestingly, in a plastome phylogenetic tree, the clade of *P. amphibia* var. *stipulacea* includes some individuals which have long, cylindrical inflorescence, ovate leaves, and tall plant height that are the hallmarks of the other American element, *P. coccinea* (**Figure 2**). This unexpected merging raises intriguing inquiries regarding the delineations between these two American entities. When two chloroplast regions were used to look at multiple individuals from 12 populations, all putative hybrid populations except Sandy Creek had the fragment types of *P. amphibia* var. *stipulacea* (**Supplementary Figure 3**). These populations seem to be of hybrid origin based on their phenotypes and contradicting plastome sequences (**Tables 2**, **3**; **Figure 3**). Although multiple individuals with *P. coccinea*-like phenotypes from those populations were sequenced for the complete plastome sequences, they had *P. amphibia* var. *stipulacea*-like genomes (**Table 3**; **Figures 2**, **3**). In addition, individuals that phenotypically resemble *P. amphibia* var. *stipulacea* also exist in these populations with flared ocreae when stranded (**Figure 3E**). However, unlike typical *P. amphibia* var. *stipulacea*, the aquatic forms did not grow far from the water’s edge, preferring to root at the water-soil border (**Figures 3A, C, G**). This behavior further strengthens the case for their hybrid origin. Thus, all four hybrid populations might have experienced hybridization between the two elements, with *P. amphibia* var. *stipulacea* likely playing as the maternal parent.

The clade of *P. coccinea* contains one putative hybrid individual (SC2), which morphologically resembles *P. coccinea*, from the Sandy Creek population. As mentioned above, in contrast to the other hybrid populations with one type of plastome, the Sandy Creek population held two different types of plastomes (**Supplementary Figure 3**). The complete plastome sequences from four individuals revealed an intriguing story: SC1, which looked like *P. coccinea*, had a *P. amphibia* var. *stipulacea*-like plastid genome. SC3 and SC4, on the other hand, showed a typical morphology of *P. amphibia* var. *stipulacea* with a *P. amphibia* var. *stipulacea*-like plastid genome. However, SC2 resembled *P. coccinea* morphologically and had a *P. coccinea*-like plastome (**Figure 2**; **Table 3**).

When the compositions of ITS2 haplotypes were examined, it was found that two individuals (SC1, SC3) had ITS2 haplotypes that closely resembled those of *P. coccinea* obtained from northern populations, PD and BL (**Table 3**). Interestingly, SC2 had ITS2 haplotypes that were similar to those of *P. coccinea* from the southern regions, RL and VF. Nevertheless, SC4 contained two distinct ITS2 haplotypes of *P. amphibia* var. *stipulacea*. These findings indicate that all three forms – the two parent species and their hybrid offspring – thrive together in the Sandy Creek population. However, a *P. coccinea*-like individual (SC2) may not be considered a parent species due to its different ITS2 haplotypes compared to putative hybrids (SC1, SC3). Thus, comprehensive study is warranted to investigate the species composition and population structure within this “hybrid swarm”, which serves as a tangible example of ongoing evolutionary processes. Furthermore, given its specific geographical location adjacent to Lake Ontario, it is critical to thoroughly investigate the extent of these hybrid swarms near Lake Ontario and along the St. Lawrence River.

## 5 Conclusions

Recognition of accurate species identity is crucial for establishing conservation programs aimed at preserving unique genetic resources. While morphological variation often presents challenges in species determination due to its continuous nature, molecular tools offer a more reliable approach. In this study, we employed whole plastome sequences and ITS2 haplotype composition to differentiate species within the *P. amphibia* complex, focusing on two American elements. Our findings support the recognition of *P. amphibia* and *P. coccinea* as distinct species within sect. *Amphibia*, while also highlighting significant genetic divergence among four geographic elements. Despite the challenges posed by the polyploid nature of the species in traditional sequencing methods, NGS enabled us to successfully determine both ITS2 haplotype composition and whole plastome sequences of members of sect. *Amphibia*. However, it is worth noting that this study utilized only one representative individual per population and focused on one nuclear region. Therefore, further investigation is necessary to explore population structure, utilizing a broader sample size and examining additional genomic features.

## Data availability statement

The datasets presented in this study can be found in online repositories. The names of the repository/repositories and accession number(s) can be found below: https://www.ncbi.nlm.nih.gov/, PRJNA1093078 and https://figshare.com/articles/dataset/Phylogenetic_analysis_using_plastid_genome_sequences/23668770.

## Author contributions

GB: Formal analysis, Investigation, Writing – review & editing. AN: Formal analysis, Investigation, Writing – review & editing. YH: Formal analysis, Investigation, Writing – review & editing. MK: Investigation, Writing – review & editing. M-JY: Conceptualization, Data curation, Formal analysis, Funding acquisition, Investigation, Methodology, Project administration, Resources, Supervision, Validation, Visualization, Writing – original draft, Writing – review & editing.
