# Supplementary material for: Evolutionary unraveling: new insights into the Persicaria amphibia complex
Source: Front Plant Sci. 2024 Jun 26;15:1408810. doi: 10.3389/fpls.2024.1408810 (PMC11233751; doi:10.3389/fpls.2024.1408810)
Supplement: Supplementary file 1 [file DataSheet_1.pdf]

Supplementary Materials

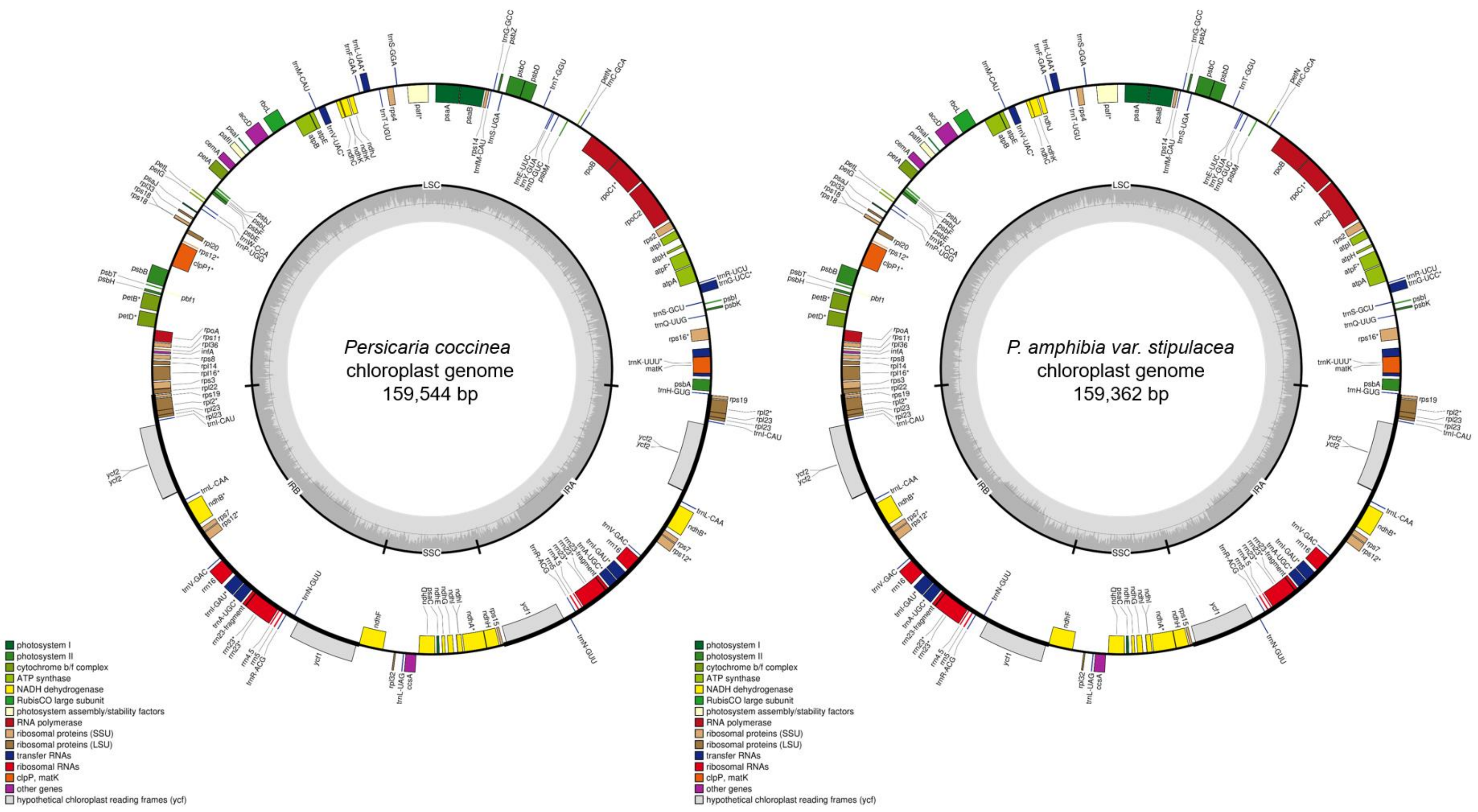

Figure S1. The structure of plastid genome of *P. coccinea* (= *P. amphibia* var. *emersa*) from Potsdam population (left) and *P. amphibia* var. *stipulacea* from Watertown (right). Two representative plastome structures were shown, as no structural variations were observed among the individuals investigated here.

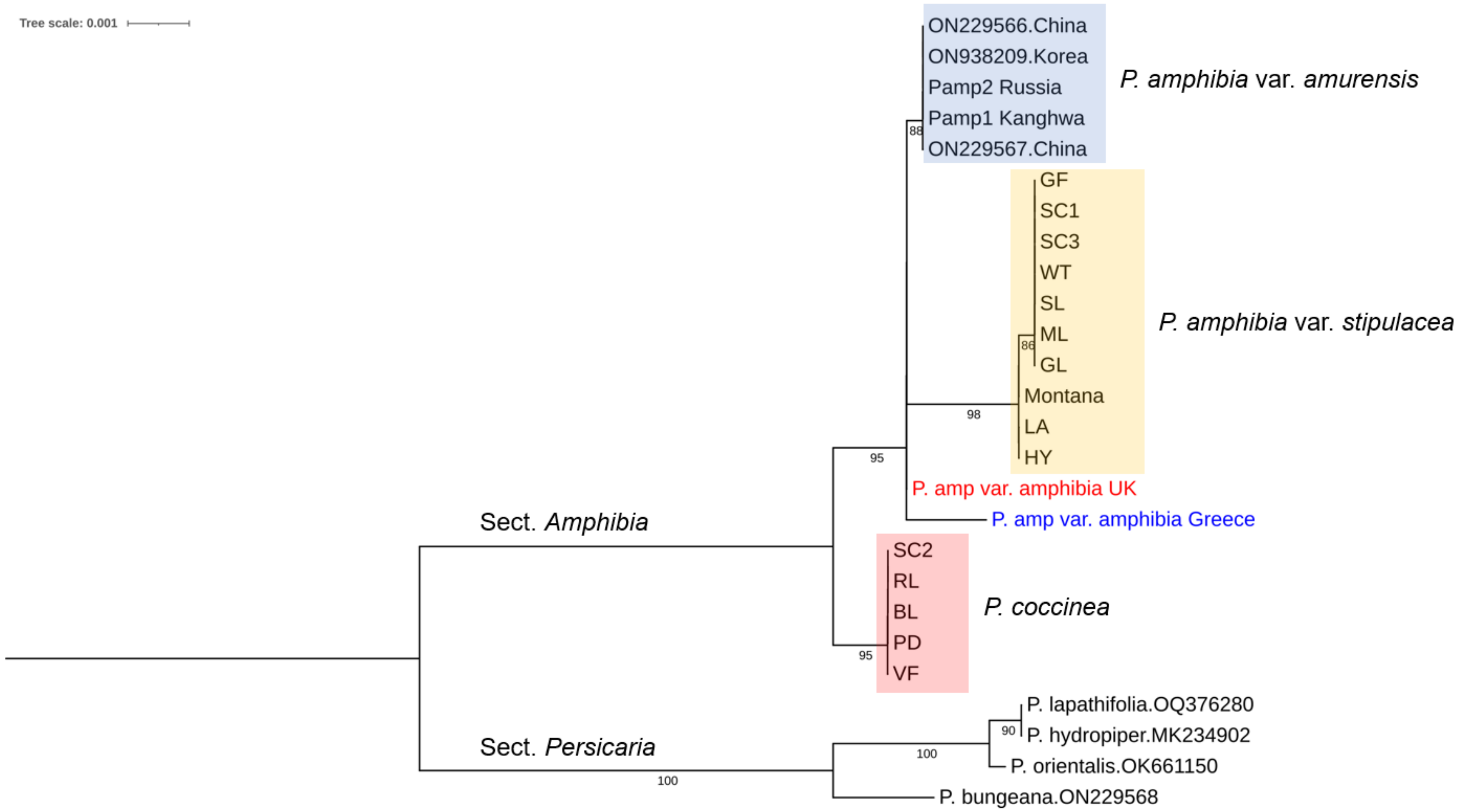

Figure S2. A maximum-likelihood tree inferred from the partial chloroplast sequences with the best fit model of K3Pu+F. The sequences of Greece individual were obtained from Gitsopoulos *et al.* (2013)'s study. The aligned length was 3,897 bp, of which 97.7% (3,808 bp) were constant across taxa and 79 sites were parsimony informative. The number below the branch shows the bootstrap support value. Two European elements (blue and red text) failed to form one clade.

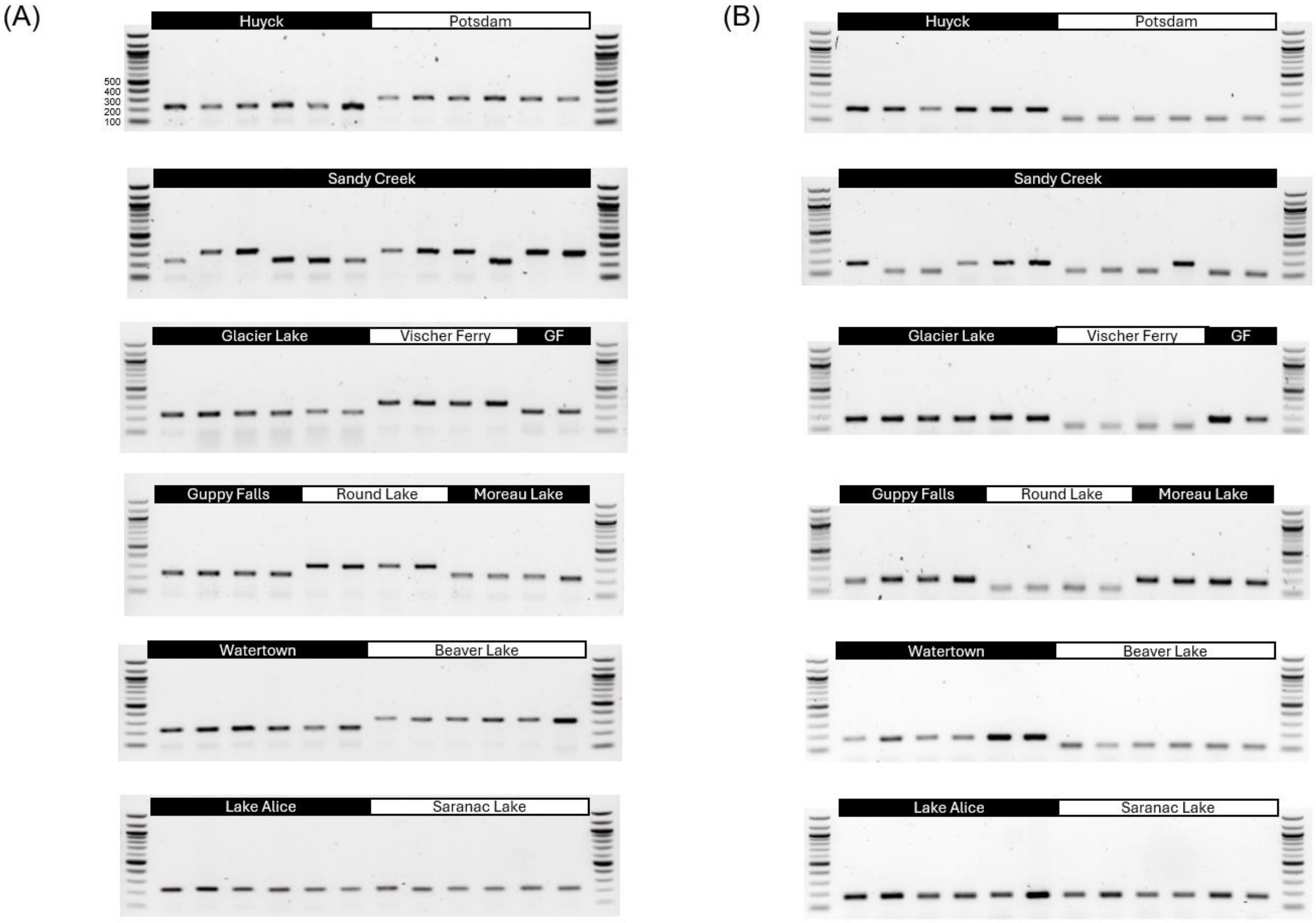

Figure S3. Genotyping results using two chloroplast regions. (A) *rpl20-rpl12* intergenic spacer (240 in *P. amphibia* var. *stipulacea* vs. 311 bp in *P. coccinea*), (B) *rpl33-rps18* intergenic spacer (171 in *P. amphibia* var. *stipulacea* vs. 108 bp in *P. coccinea*). Note that Sandy Creek population shows co-occurrence of two chloroplast genomes.

Table S1. Seven chloroplast regions which show differences between two American elements. Asterisk (*) indicates the chloroplast regions used in genotyping each population.

Chloroplast region	Primer sequences (5’->3’)	Amplicon size (bp) in *P. coccinea*	Amplicon size (bp) in *P. amphibia* var. *stipulacea*	Difference in amplicon sizes
psbA-trnK IGS	F: CAGTATAACATGACTTATATGC<br>R: TCATATCGAAGTCATATGTG	130	124	6 bp
atpF intron	F: TGCTATGGTTCTTCCATATG<br>R: CAAGAATAGACTGGATTCAAC	127	120	7 bp
rpoC1 intron	F: ACTACTCCTTACTCAAGTTC<br>R: GGAAGTAGACTACTCAAG	131	123	8 bp
trnE-trnA IGS	F: CTTGGTTCATTGAAGTGATAG<br>R: CTATGCGGATAAGCTATCTC	159	144	15 bp
trnT-trnL IGS	F: CATTCCAATCCTAACAATTAG<br>R: GATAGAACTCATAATTGAATTCA	172	148	24 bp
rpl20-rpl12 IGS*	F: TCGAACGTCTGTATATCAC<br>R: AGATCCGACCGAAATAGG	311	240	71 bp
rpl33-rps18 IGS*	F: TCATACGCAATAGATGAATATG<br>R: GCCGAGGAACATGTACTAG	108	171	63 bp
